# Current Status and Future Projections of Artificial Intelligence–Assisted Ultrasonography and Needle Visibility Methods in Regional Anesthesia

**DOI:** 10.5152/eurasianjmed.2026.261453

**Published:** 2026-04-24

**Authors:** Yasin Tire, Aydın Mermer, Mustafa Aydemir, Ömer Keklicek, Muhammed Nezih Koç, Mehmet Akif Yazar

**Affiliations:** 1 Department of Anesthesiology and Reanimation, Konya City Hospital, University of Health Sciences, Konya, Türkiye; 2 Outcomes Research Consortium, Houston, Texas, USA

**Keywords:** Artificial intelligence, deep learning, medical imaging, needle visualization, ultrasound-guided regional anesthesia

## Abstract

Ultrasound-guided regional anesthesia (UGRA) has revolutionized regional anesthesia by enabling direct visualization of neural structures, surrounding anatomy, and local anesthetic spread. However, consistent needle visualization remains challenging due to anisotropy, steep insertion angles, tissue deformation, and ultrasound artifacts, potentially increasing procedural difficulty and the risk of complications such as vascular puncture, pneumothorax, or intraneural injection.

Recent advances in artificial intelligence (AI) offer promising solutions. Artificial intelligence–assisted ultrasound systems using deep learning and convolutional neural networks can perform real-time anatomical segmentation, automated needle tracking, and image optimization. These platforms highlight nerves, vessels, and fascial planes with color overlays, guide needle trajectory, and provide feedback on image quality and probe positioning.

In addition to procedural assistance, AI may improve training by accelerating anatomical recognition and reducing inter-operator variability. Nevertheless, concerns persist regarding automation bias, algorithm performance in atypical anatomy, and the necessity of ongoing clinician oversight.

Overall, AI-assisted ultrasonography represents a significant step toward safer, more standardized, and potentially more efficient regional anesthesia practice.

**Main Points**

- Mitigating cognitive barriers: Artificial intelligence (AI) serves as an augmented cognitive layer or “digital second observer,” helping to bridge the interpretation gap and reduce “inattentional blindness” during complex needle-to-target maneuvers.
- Overcoming physical limitations: Advanced deep learning architectures and convolutional neural networks can filter acoustic noise and “triangulate” the true needle tip position, addressing physical challenges like anisotropy and tissue deformation that often obscure visualization in deep nerve blocks.
- Standardizing clinical excellence: By providing real-time anatomical segmentation and automated color-coded masking of nerves and vessels, AI-assisted systems aim to standardize the procedural “standard of care” and narrow the performance gap between novice and expert practitioners.
- Proactive safety identification: AI technology enhances patient safety by persistently flagging “no-go zones,” such as the pleura or peritoneum, and providing predictive trajectory corridors to prevent iatrogenic injuries during high-risk blocks.
- Educational and quality transformation: The integration of AI accelerates the learning curve through immediate high-fidelity feedback and enables objective post-procedural audits based on measurable metrics rather than just the number of blocks performed.

## Introduction and Historical Perspective

The integration of ultrasonography (US) into the clinical workflow of regional anesthesia is not merely a technical update; it is widely regarded as a fundamental paradigm shift and a definitive revolution in the field of anesthesiology.^1^^,^^2^ For the greater part of the twentieth century, regional anesthesia was practiced as a “blind” art, relying heavily on a clinician’s mastery of surface anatomical landmarks and the interpretation of indirect tactile feedback. Techniques such as the “loss of resistance” or the characteristic “click” felt as a needle traversed distinct fascial planes were the primary indicators of needle position. The later introduction of peripheral nerve stimulation (PNS) added a functional dimension to this process, allowing clinicians to elicit motor responses to confirm proximity to a nerve.^3^

However, these traditional methods were inherently fraught with limitations. The reliance on surface landmarks failed to account for the vast spectrum of anatomical variability factors such as high body mass index (BMI), previous surgeries, or congenital anomalies often rendered these markers unreliable. Furthermore, PNS, while functional, provided only an indirect confirmation of nerve localization; it could not visualize the relationship between the needle tip and adjacent vulnerable structures like blood vessels or the pleura. Consequently, these “blind” eras were characterized by inconsistent block success rates, a higher incidence of systemic local anesthetic toxicity due to accidental intravascular injection and a persistent risk of nerve injury from mechanical trauma.^4^

The advent of ultrasound-guided regional anesthesia (UGRA) fundamentally disrupted this landscape. By providing a real-time “window” into the body, US allowed for the direct visualization of target neural structures, surrounding vascular anatomy, and perhaps most critically the dynamic monitoring of local anesthetic spread within the targeted fascial planes.^5^^,^^6^ This visual confirmation transitioned the field from a probabilistic practice to one of anatomical certainty. Large-scale clinical trials and meta-analyses have consistently demonstrated that UGRA significantly improves block onset times, increases success rates, and allows for a substantial reduction in the minimum effective volume of local anesthetic, thereby enhancing the overall safety profile of the procedure.^7^

The technology provides the image, but the human brain must interpret it. This introduces a “cognitive gap,” where even experienced clinicians can fall victim to “inattentional blindness” focusing so intently on the advancing needle that they fail to recognize a nearby vessel or the pleura. The technical difficulty of maintaining the needle within the thin ultrasound beam, especially during deep blocks or in patients with challenging habitus, remains a primary cause of block failure and iatrogenic complications like pneumothorax or intraneural injection.^8^

In this context, artificial intelligence (AI) represents the next logical evolutionary step in regional anesthesia. By leveraging advanced deep learning architectures and convolutional neural networks (CNNs), AI systems can process ultrasound data with a level of granularity and speed that transcends human perception. Rather than replacing the clinician, AI acts as an augmented cognitive layer, a digital “second observer” that never tires or loses focus. These systems are designed to bridge the gap between image acquisition and clinical action, offering automated anatomical segmentation, real-time needle tracking, and predictive safety alerts.^9^ Entering this new era, the goal of AI integration is to standardize excellence, ensuring that the precision of the expert becomes the baseline for every practitioner (Table 1).

### Technical and Physical Challenges in Needle Visibility

The clinical efficacy of UGRA is inextricably linked to the operator’s ability to visualize the needle in relation to the target anatomy. Unlike the visualization of organic tissues, needle visibility is governed by the complex interplay of ultrasound physics, specifically the principles of specular reflection and diffuse scattering.^3^^,^^10^ When the ultrasound beam strikes a smooth, metallic surface like a needle, the majority of the energy is reflected back. However, the quality of this returning signal is highly dependent on the “angle of incidence.” In an ideal scenario, the needle is perpendicular (90°) to the beam, allowing for maximum signal return. In clinical reality, achieving this “golden angle” is often physically impossible, leading to a cascade of technical challenges that compromise patient safety^11^ (Table 2).

### Anisotropy and the “Deep Block” Dilemma

The most significant physical hurdle in UGRA is anisotropy. This phenomenon occurs when the ultrasound beam hits a structure at an oblique angle, causing the reflected echoes to scatter away from the transducer rather than returning to it. In the context of needle guidance, as the insertion angle becomes steeper moving away from the horizontal plane, the needle’s echogenicity diminishes exponentially. This is not a linear loss; beyond a critical angle (typically 45-60 degrees), the physics of reflection dictates that almost no signal returns to the probe.^12^

This challenge is most acutely felt during the performance of deep nerve blocks, such as the transmuscular quadratus lumborum block, psoas compartment block, or the sciatic nerve block in the subgluteal space. In these scenarios, the target is located deep beneath thick layers of adipose and muscular tissue, necessitating a steep needle trajectory. As the angle of the needle increases to reach these depths, the needle shaft, and more critically, the needle tip, often vanish from the real-time display.

**Figure 1:**
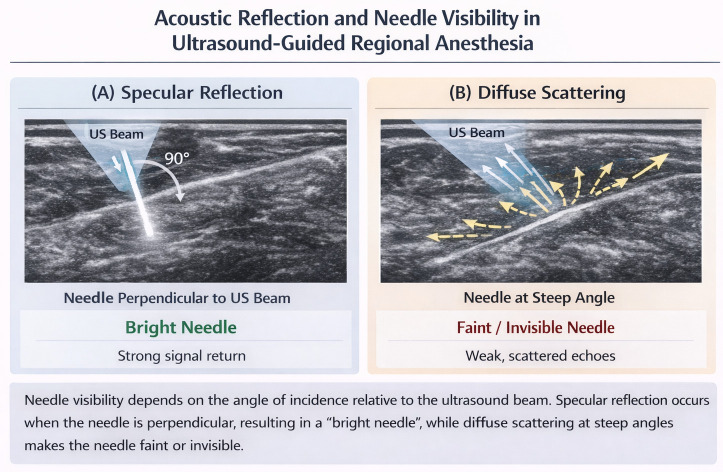
Acoustic reflection mechanisms and their impact on needle visibility during ultrasound-guided regional anesthesia.

Recent literature, including specialized reviews (e.g., IJA 2024), emphasizes that even “enhanced” echogenic needles, which feature laser-etched dimples to increase scattering, have physical limits when dealing with extreme anisotropy.^13^ Furthermore, in patients with a high BMI, the ultrasound beam undergoes significant attenuation and scattering before it even reaches the needle, burying the already weak needle signal under a layer of “speckle noise.” Traditional compensatory maneuvers, such as “rocking” or “heel-toe” probe adjustments, attempt to bring the beam back to a 90-degree alignment, but these maneuvers often distort the anatomical view of the nerve, creating a paradox: the clinician can either see the nerve clearly or the needle clearly, but rarely both at once in deep trajectories^14^ (Figure 1).

### Tissue Deformation, Motion Artifacts, and “The Ghost Tip”

As a needle penetrates biological tissue, it does not move through a static medium. Instead, it causes significant dynamic tissue deformation. The displacement of fascial planes and the compression of adipose tissue create a visual “shimmer” or “tenting” effect. For many clinicians, particularly those in the early stages of their learning curve, this tissue movement is often misinterpreted as the needle tip itself—a dangerous phenomenon known as the “pseudo-tip” or “ghost tip” artifact.^15^

Beyond simple deformation, several physical artifacts complicate the clinical image:

- Acoustic shadowing: The dense metallic structure of the needle may block the ultrasound beam entirely, casting a dark shadow over the very structures (vessels or nerves) the clinician needs to monitor.
- Reverberation artifacts: Multiple reflections between the needle and the transducer can create a “comet-tail” appearance or multiple parallel lines (ghost needles), which can lead to catastrophic errors in depth perception and unintended needle advancement.^16^
- Side-lobe artifacts: These can cause the needle to appear in an incorrect anatomical location on the screen, potentially leading to unintentional puncture of adjacent vital structures.

### The Role of Artificial Intelligence in Filtering Physical Noise

In this technically hostile environment, AI algorithms, particularly those utilizing pixel-level segmentation, offer a solution that transcends manual probe manipulation. While the human eye may struggle to distinguish a weak reverberation artifact from the true metallic tip in a grainy image, a CNN can be trained to recognize the specific “digital signature” of a needle based on temporal and spatial data.^17^

Artificial intelligence–assisted systems can analyze the frame-by-frame deformation of tissue to mathematically “triangulate” the most likely position of the needle tip. By filtering out acoustic noise and enhancing the contrast of the hyperechoic needle shaft, AI transforms a cluttered, artifact-heavy image into a clean, actionable map. This capability is particularly vital for out-of-plane (OOP) techniques, where the needle appears only as a single dot. Artificial intelligence can differentiate this “dot” from other bright spots in the tissue (such as small calcifications or thick fascia), providing the clinician with the certainty that the visualized point is indeed the needle tip before any drug is injected.^18^

### Technical Analysis of Artificial Intelligence–Assisted Technologies

The integration of AI into the regional anesthesia workflow is not a monolithic development but rather a convergence of several computational disciplines. Modern AI applications are built upon 3 primary pillars that address the specific limitations of human perception: anatomical segmentation, dynamic needle tracking, and intelligent image optimization.^18^^,^^19^ By synthesizing these elements, AI transforms a standard ultrasound machine from a passive display monitor into an active intraoperative consultant.

### Real-Time Anatomical Segmentation and Cognitive Mapping

The cornerstone of AI in regional anesthesia is the ability to perform high-fidelity anatomical segmentation in real-time. Systems like ScanNav Anatomy PNB (intelligent ultrasound) and NerveSIGHT are developed using deep learning architectures—specifically U-Net or similar encoder-decoder networks—trained on massive, expert-annotated ultrasound datasets containing hundreds of thousands of frames across diverse patient demographics.^20^

These systems automatically identify and distinguish complex tissue architectures, including fascial planes, peripheral nerves, and vascular structures. To bridge the “interpretation gap” for the clinician, the AI overlays intuitive, color-coded masks directly onto the live grayscale image:

- Nerve structures: Typically highlighted in yellow, allowing for immediate identification of the target, even when the nerve is iso-echogenic relative to surrounding tendons.
- Vascular anatomy: Arteries and veins are flagged in red and blue, providing a continuous “Doppler-like” awareness without the need for manual color flow activation, which can sometimes degrade grayscale resolution.^21^
- Fascial compartments: Often highlighted in cyan or purple, these masks guide the clinician toward the correct plane for plane blocks (e.g., erector spinae plane (ESP) or Pectoralis (PECS) blocks I & II).

Furthermore, the clinical significance of segmentation extends beyond just finding the target; it is equally about identifying “no-go zones.” Artificial intelligence can proactively flag critical boundaries, such as the pleura in supraclavicular or thoracic blocks, or the peritoneum in abdominal wall blocks. By providing a persistent visual alert of these structures, AI reduces the cognitive load on the anesthesiologist and acts as a digital safety net against iatrogenic injury.^22^

### Dynamic Needle Tracking and Trajectory Prediction

While anatomical recognition is the first step, the primary cause of complications remains the loss of needle-tip visualization. Artificial intelligence addresses this through sophisticated object detection and tracking algorithms. Unlike basic software that simply brightens hyperechoic lines, AI-driven tracking identifies the unique “metallic signature” and the temporal movement of the needle.^16^

One of the most transformative features of this pillar is trajectory prediction. By analyzing the current orientation of the needle shaft, predictive algorithms can extrapolate the needle’s path and display a projected “safety corridor” toward the target. This allows the clinician to correct the needle angle before it enters a hazardous area, significantly reducing trial-and-error punctures and minimizing unnecessary tissue trauma.

**Figure 2.**
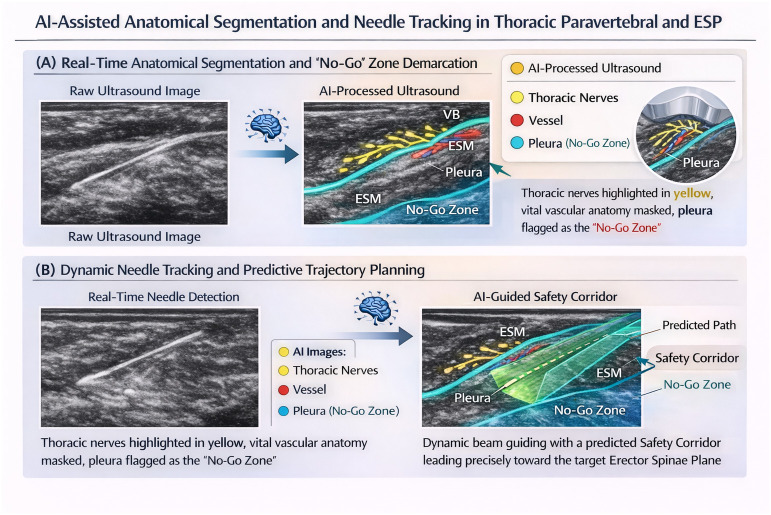
AI-Assisted Anatomical Segmentation and Needle Tracking in Thoracic Blocks. ** (A) Real-Time Anatomical Segmentation and ‘No-Go’ Zone Demarcation. ** * This panel demonstrates the transition from a raw greyscale ultrasound image to an AI-processed view.

The challenge of OOP techniques is also addressed. In OOP, the needle appears only as a transverse cross-section (a single dot). Artificial intelligence can differentiate this dot from other “bright” tissue artifacts by analyzing localized tissue deformation patterns. When the needle moves, the AI detects the specific way the surrounding fascia “tents” or displaces, confirming the true location of the needle tip with a degree of precision that manual observation often lacks^23^ (Figure 2).

### Image Quality Indexing and Interactive Operator Guidance

As emphasized in recent literature (e.g., APM 2025), AI is evolving from a passive viewer into an active guide. The quality of an AI’s output is inherently limited by the quality of the input image—the “garbage in, garbage out” principle. To counter this, advanced systems now include image quality indexing.

These systems continuously monitor the live feed for parameters such as:

- Acoustic coupling: Detecting if there is insufficient gel or uneven probe pressure.
- Gain and depth settings: Determining if the target structure is too dark or too deep for optimal segmentation.
- Anisotropy detection: Recognizing when the probe angle is suboptimal for visualizing the nerve’s internal architecture.^24^

When image quality falls below a reliable threshold, the AI does not just fail; it provides real-time corrective feedback. For instance, the system might display an on-screen prompt instructing the operator to “Tilt the probe cranially” or “Increase pressure on the lateral edge.” This interactive guidance is particularly beneficial in educational settings and for non-expert users, ensuring that the procedural “standard of care” is maintained regardless of the operator’s baseline skill level.^25^

### Educational Impact and Expanded Clinical Outcomes

The integration of AI into clinical practice represents more than a technical refinement; it is a fundamental transformation of how regional anesthesia is taught, practiced, and audited. By providing a real-time, objective validation of anatomy and technique, AI addresses the inherent subjectivity that has historically characterized anesthesia training.^25^

### Shortening the Learning Curve and Cognitive Acceleration

Traditional ultrasound education is a time-intensive process that requires the development of complex visuospatial skills. Learners must transition from “looking” at a grayscale screen to “seeing” the anatomical relationships—a process that typically requires hundreds of supervised repetitions. Artificial intelligence accelerates this pattern recognition by providing immediate, high-fidelity feedback.

Data from recent reviews (e.g., IJA 2024) suggest that AI-assisted US may facilitate faster identification of key anatomical landmarks compared with conventional ultrasound training methods.^24^^,^^26^ This “cognitive offloading” allows the student to focus less on the struggle of identification and more on the nuances of needle manipulation and patient safety. Furthermore, AI acts as a 24/7 tutor in resource-limited environments or during late-night shifts when expert supervision may be unavailable, ensuring that the quality of education does not fluctuate with staff availability.^25^

### Standardization and the Management of Anatomical Variation

A defining characteristic of an “expert” anesthesiologist is the ability to recognize and adapt to anatomical variations. For a novice, a bifid median nerve or an aberrant vascular path can lead to confusion, block failure, or inadvertent injury. Artificial intelligence systems, trained on diverse global datasets, are uniquely equipped to identify these “outlier” anatomies that a human operator might see only a few times in a career.^27^

By highlighting these variations in real-time, AI standardizes the “safety margin” across the department. It effectively narrows the performance gap between the novice and the expert, ensuring that block success is dictated by the patient’s needs rather than the luck of having an experienced operator on duty. This democratization of expertise is a cornerstone of modern perioperative safety initiatives, aiming for a “zero-harm” environment in regional anesthesia.^28^

### Diaphragm Function and Procedure-Specific Safety

The clinical impact of AI is perhaps most visible in high-risk blocks where the margin for error is measured in millimeters. Two critical areas where AI enhances safety are the preservation of respiratory function and the prevention of pleural injury.

- Phrenic nerve and diaphragm protection: During interscalene brachial plexus blocks, the proximity of the phrenic nerve often leads to unintended blockade, resulting in hemi-diaphragmatic paralysis. This can be catastrophic for patients with borderline respiratory reserve. Artificial intelligence systems can be trained specifically to track the phrenic nerve as it courses over the anterior scalene muscle, alerting the clinician to maintain a safe “distance of injection.”^23^
- Pleural boundary visualization: In thoracic blocks such as the serratus anterior plane or ESP blocks, the primary risk is pneumothorax. Artificial intelligence–enhanced visualization provides a persistent, high-contrast marker for the pleura. By flagging this “critical boundary,” AI allows the clinician to perform these blocks with greater confidence, potentially expanding the use of regional techniques in patients where the risk of lung injury was previously deemed too high.^22^

### Post-Procedural Audit and Quality Improvement

Beyond the immediate procedure, AI-assisted technologies facilitate a new era of quality assurance. Because these systems can automatically log “time-to-identification” of nerves and “needle-tip visibility duration,” departments can generate objective performance metrics for their staff. This data-driven approach allows for targeted remedial training and the objective assessment of clinical competency, moving away from the arbitrary “number of blocks performed” as a metric for proficiency.^29^

## Discussion: Current Limitations and Future Vision

The transition of AI from a promising research concept to a mainstay of the clinical operating room is one of the most significant challenges in modern anesthesiology. While the potential for improved safety and efficiency is undeniable, the medical community must navigate a complex landscape of technological, ethical, and practical hurdles before AI becomes an invisible, seamless part of the perioperative workflow.^30^

### Technological and Ethical Constraints: The Human-Machine Interface

A primary concern discussed in current literature is the risk of automation bias or overreliance. There is a tangible danger that clinicians, particularly novices, may develop a “false sense of security,” delegating their critical thinking to the AI algorithm. If a clinician stops verifying the AI’s anatomical labels against their own knowledge, they may overlook a “false-positive” identification—where the AI incorrectly labels a vessel as a nerve—leading to catastrophic errors.^30^

Furthermore, AI systems are only as robust as the data on which they were trained. “Out-of-distribution” scenarios—such as patients with massive tumors distorting anatomy, severe post-traumatic scarring, or rare congenital anomalies—can cause the algorithm to fail in unpredictable ways. Therefore, the prevailing consensus in recent reviews (e.g., APM 2025) is that AI must remain a decision-support tool rather than a decision-maker. The final “go/no-go” judgment must always reside with the human clinician, who maintains legal and ethical accountability for the patient’s outcome.^31^

### Multimodal Sensor Integration and Emerging Innovations

The “Future Projections” of regional anesthesia involve moving beyond simple grayscale ultrasound. With the advent of of multimodal imaging, AI synthesizes data from various sensor types to provide a holistic view of the procedure.^32^

- Thermal imaging integration: Future AI systems could integrate real-time thermography to map the distribution of analgesia. By detecting the sympathetic-mediated temperature changes in the limb following a successful block, AI could provide objective confirmation of block success minutes before clinical sensory changes occur.
- Pressure-aware imaging: One of the most promising innovations is the development of smart, pressure-sensitive sterile probe covers. These covers, aligned with emerging internet of things concepts, can provide the AI with data on the exact force being applied to the patient’s skin. Since probe pressure significantly alters tissue echogenicity and anatomical depth, “pressure-aware AI” could guide the operator to use the optimal force needed to visualize deep structures without compressing the very veins they are trying to avoid.^33^
- Augmented reality (AR): The integration of AI with AR headsets (like Microsoft HoloLens or specialized medical AR) could project the AI-annotated “no-go zones” and the projected needle trajectory directly onto the patient’s skin, allowing the anesthesiologist to maintain a natural line of sight without constantly looking away at a monitor.

### Toward Autonomous Regional Anesthesia: The Robotic Frontier

The long-term vision for the field involves the synergy of AI and robotic assistance. While the idea of a fully autonomous robot performing a nerve block may seem futuristic, the foundations are already being laid. Robotic and AI-assisted systems are increasingly being explored for autonomous ultrasound acquisition and interventional procedures. Artificial intelligence yokes can deliver needles along “optimal safety trajectories” with a level of steadiness and precision that no human hand can match.^30^

These systems could prove transformative in:

- Remote/resource-limited settings: Allowing a generalist or a technician to perform advanced regional blocks under the remote supervision of a specialist, with the AI and robot ensuring the needle never touches a critical structure.
- Battlefield or disaster medicine: Providing high-quality pain relief in environments where specialist anesthesiologists are not immediately available.

## Conclusion

Artificial intelligence-assisted US and needle visualization represent more than just a technical convenience; they signify a major step toward eliminating human error in regional anesthesia. By addressing the fundamental physical limitations of ultrasound, such as anisotropy and tissue deformation, and by shortening the learning curve for the next generation of anesthesiologists, AI is redefining the “standard of care.”

The current literature consistently suggests that AI will evolve from an optional, high-tech enhancement into a standard, mandatory component of future practice—much like the pulse oximeter or the ultrasound machine itself. Beyond the technical benefits of anatomical segmentation and needle tracking, these systems establish a new paradigm centered on patient safety, procedural precision, and the democratization of medical expertise. As we look toward 2030 and beyond, the collaboration between the augmented cognitive layer of AI and the clinical intuition of the anesthesiologist promises to make regional anesthesia safer and more accessible than ever before.

## Figures and Tables

**Table 1.** Comparative Evolution of Regional Anesthesia Techniques: From Landmarks to AI

**Feature**	**Traditional Methods (Landmark/PNS)**	**Standard UGRA (Gold Standard)**	**AI-Assisted UGRA (Next Generation)**
Localization method	Blind/indirect (tactile feel or motor response)	Direct real-time visualization (grayscale)	Augmented visualization (color-coded masking)
Needle tip tracking	Not possible (assumed based on clicks/twitches)	Manual/operator-dependent (subject to anisotropy)	Automated/predictive (AI-enhanced trajectory)
Anatomical recognition	Conceptual (mental mapping of surface landmarks)	Human-dependent (pattern recognition required)	Machine-assisted (automated segmentation of nerves/vessels)
Safety mechanisms	Limited (high risk of LAST and neuropathy)	Operator vigilance (risk of inattentional blindness)	Digital “Second Observer” (real-time safety alerts)
Learning curve	Extremely long (steep apprenticeship model)	Moderate to long (visuospatial skill acquisition)	Accelerated (AI-assisted landmark recognition and pattern identification)
Standardization	Low (high inter-operator variability)	Moderate (dependent on technical expertise)	High (standardized safety margins across experience levels)

AI, artificial intelligence; LAST, local anesthetic systemic toxicity; PNS, peripheral nerve stimulation; UGRA, ultrasound-guided regional anesthesia.

**Table 2.** Commercial and Emerging AI-Assisted Systems in Regional Anesthesia

**System Name**	**Developer**	**Core AI Pillar**	**Target Anatomy/Blocks**	**Clinical Key Feature**
ScanNav Anatomy PNB	Intelligent Ultrasound	Anatomical segmentation	10 common peripheral nerve blocks (e.g., Interscalene, Femoral, ESP)	Real-time color-coded overlays (Yellow: nerve, Red/Blue: vessels, Cyan: fascia).
NerveSIGHT	Philips/Deep01	Nerve localization	Brachial plexus and major peripheral nerves	High-fidelity neural segmentation optimized for high-resolution transducers.
NeedleSmart	NeedleSmart Ltd	Needle tracking	Deep blocks and OOP techniques	Trajectory prediction and real-time needle-tip highlighting to prevent overshoot.
Ezono / AI-Assist	eZono AG	Needle visualization	General PNB and vascular access	Focuses on reducing anisotropy effects through signal enhancement of the needle shaft.
IQ Indexing (Prototype)	Academic/Research	Image optimization	Universal UGRA	Active operator guidance providing real-time prompts to adjust gain, depth, and probe pressure.

AI, artificial intelligence; AR, augmented reality; CNN, convolutional neural network; ESP, erector spinae plane; IQI, image quality indexing; OOP, out-of-plane; PNB, peripheral nerve block; UGRA, ultrasound-guided regional anesthesia.

## Data Availability

The data that support the findings of this study are available on request from the corresponding author.
